# Incidência de lesões musculoesqueléticas em gramados natural e sintético na Série A do Campeonato Brasileiro de Futebol de 2024: Um estudo retrospectivo

**DOI:** 10.1055/s-0046-1822978

**Published:** 2026-07-28

**Authors:** Matheus Martins Godoy, Jéssica Chávare, Pedro Lucas Leal Chaves, João Pedro Ferreira de Almeida, Matheus Alves de Carvalho Freitas, Gabriel Moraes de Oliveira, Flávia Costa Oliveira Magalhães, Israel Teoldo da Costa

**Affiliations:** 1 Centro Universitário de Patos de Minas (UNIPAM), Patos de Minas, MG, Brazil; 2 Universidade Professor Edson Antônio Velano (UNIFENAS), Belo Horizonte, MG, Brazil; 3 Centro Universitário de Belo Horizonte (UNIBH), Belo Horizonte, MG, Brazil; 4 Universidade Federal do Recôncavo da Bahia (UFRB), Santo Antônio de Jesus, BA, Brazil; 5 Massachusetts General Hospital, Harvard Medical School, Boston, MA, United States; 6 Escola Paulista de Medicina, Universidade Federal de São Paulo, São Paulo, SP, Brazil; 7 Universidade Federal de Minas Gerais (UFMG), Belo Horizonte, MG, Brazil; 8 Núcleo de Pesquisa e Estudos em Futebol (NUPEF), Departmento de Educação Física, Universidade Federal de Viçosa (UFV), Viçosa, MG, Brazil

**Keywords:** esportes, futebol, joelho, sistema musculoesquelético/lesões, tornozelo, trauma, ankle, knee, musculoskeletal system/injuries, soccer, sports, trauma

## Abstract

**Objetivo:**

Descrever e comparar partidas disputadas em gramado sintético e gramado natural na Série A do Campeonato Brasileiro de Futebol, com foco específico na incidência e na distribuição observadas de lesões musculoesqueléticas.

**Métodos:**

Neste estudo observacional e retrospectivo, foram analisados todos os 380 jogos da temporada de 2024. Os dados sobre o tipo de gramado, as características das partidas e as lesões foram obtidos exclusivamente de fontes secundárias acessíveis ao público, como o *site* oficial da Confederação Brasileira de Futebol (CBF; https://www.cbf.com.br ) e os principais meios de comunicação nacionais. Foram excluídos casos com informações conflitantes. A exposição durante as partidas foi estimada, presumindo-se 22 jogadores e 90 minutos, com a incidência expressa como lesões por mil horas-jogador. Subanálises excluíram relesões anteriores e casos de trauma direto.

**Resultados:**

Um total de 88 lesões foi registrado, o que indica 6,6 por mil horas-jogador em gramado natural, em comparação com 9,5 por mil horas-jogador em gramado sintético. As lesões nos isquiotibiais e as entorses de tornozelo foram os tipos mais frequentemente observados. Quanto aos gramados, o de elastômero termoplástico ( *thermoplastic elastometer* , TPE, em inglês) apresentou as maiores taxas entre os sintéticos, ao passo que o azevém perene apresentou as maiores taxas entre os naturais. O número de lesões variou conforme o contexto, com taxas notáveis observadas em gramado sintético durante as partidas da tarde ou da noite e em temperaturas mais baixas. Os tratamentos cirúrgicos foram observados frequentemente com relação ao gramado natural, ao passo que os tempos de recuperação diferiram minimamente. Notavelmente, uma equipe cujo estádio era de gramado sintético e outra cujo estádio era de gramado natural registraram totais de lesões iguais.

**Conclusão:**

As diferenças na ocorrência de lesões entre os tipos de gramado foram mínimas. Considerando-se o desenho retrospectivo, a confiança nos dados secundários, o potencial de subnotificação e o controle limitado de fatores de confusão, os resultados representam associações observacionais. Estudos prospectivos com vigilância padronizada são necessários.

## Abstract (en)

**Objective:**

To describe and compare matches played on artificial and natural turfs during the 2024 Campeonato Brasileiro Série A (the championship of the highest level of the professional association football league in Brazil), focusing specifically on the observed incidence and distribution of musculoskeletal injuries.

**Methods:**

In the current retrospective observational study, we analyzed all 380 matches of the 2024 season. Data regarding turf type, match characteristics, and injuries were obtained exclusively from publicly available secondary sources, including the official website of Confederação Brasileira de Futebol (CBF; https://www.cbf.com.br ) and major national media outlets. Cases with conflicting information were excluded. Exposure during the match was estimated assuming 22 players and 90 minutes, with incidence expressed as injuries per 1 thousand player-hours. Sub-analyses excluded previous reinjuries and direct trauma cases.

**Results:**

A total of 88 injuries was recorded, showing 6.6 injuries per 1 thousand player-hours on natural turf versus 9.5 injuries per 1 thousand player-hours on artificial turf. Hamstring strains and ankle sprains were the most frequently observed types. Regarding the turfs, thermoplastic elastomer (TPE) turf presented the highest rates among the artificial ones, while perennial ryegrass presented the highest rates among the natural ones. Injury numbers varied by context, with considerable rates observed regarding artificial turf during afternoon or night matches and at lower temperatures. Surgical treatments were frequently observed in association with natural turf, whereas recovery times differed minimally. Notably, one team whose stadium used artificial turf home team and one team whose stadium used natural turf recorded identical injury totals.

**Conclusion:**

Differences in injury occurrence between turf types were minimal. Given the retrospective design, secondary data reliance, underreporting potential, and limited control of confounders, the findings represent observational associations. Future prospective studies using standardized surveillance are warranted.

## Introdução

O desenvolvimento do gramado sintético, amplamente utilizado no futebol mundial, começou no final da década de 1990, e, em 2004, a Federação Internacional de Futebol (Fédération Internationale de Football Association, FIFA, em francês) autorizou o uso do gramado sintético de terceira geração em partidas oficiais. 1 2 3 Apesar de sua adoção mundial, persistem preocupações quanto ao seu potencial de causar lesões graves em jogadores profissionais. Acredita-se que as lesões musculoesqueléticas resultem de fatores como o tipo e as propriedades do gramado, 2 4 5 6 7 as características do calçado, 8 9 10 o uso excessivo e o trauma de impacto, 11 12 13 todos considerados os principais contribuintes.

Revisões sistemáticas e metanálises recentes têm buscado esclarecer a relação entre a superfície de jogo e o risco de lesões. Gould et al. 6 relataram taxas gerais semelhantes de lesões nos membros inferiores na comparação entre gramados sintéticos e naturais, embora as lesões no tornozelo tenham ocorrido com maior frequência em gramados sintéticos. Da mesma forma, Kuitunen et al. 8 demonstraram incidência comparável de lesões na comparação entre gramados, somente com aumentos modestos em tipos específicos de lesões no gramado sintético. Meyers 10 não encontrou diferenças clinicamente relevantes na gravidade geral da lesão na comparação entre gramados sintéticos e naturais, apesar das variações nos mecanismos de lesão. Essas descobertas sugerem que, embora o gramado sintético possa influenciar certos padrões de lesão, seu perfil de risco geral permanece comparável ao do gramado natural.

No futebol brasileiro, o Campeonato da Série A está entre as ligas mais competitivas e prestigiadas, com clubes de ponta e atletas de elite. O gramado do jogo—natural ou sintético—influencia muito o desempenho e o risco de lesões. 14 15 16 No entanto, diferenças no clima, no congestionamento do jogo, na manutenção, além da heterogeneidade do gramado, podem limitar a aplicabilidade direta do achado internacional ao contexto profissional brasileiro. Embora as lesões sejam frequentes, evidências sugerem que o tipo de gramado afeta a incidência, com gramados sintéticos associados a maior risco de lesão traumática. 4 17 18

A temporada da Série A do Campeonato Brasileiro de 2024 forneceu um contexto ideal para analisar lesões musculoesqueléticas em diferentes gramados. A alternância entre os tipos de gramado, a programação densa de jogos e o jogo intenso podem aumentar o risco de lesões, o que enfatiza a importância de se avaliar as condições do campo e a saúde do atleta. 19 20 Portanto, este estudo teve como objetivo analisar a incidência e as características das lesões musculoesqueléticas ocorridas em gramados sintéticos e naturais durante a temporada 2024 da Série A do Campeonato Brasileiro. A hipótese investigacional foi a de que partidas disputadas em gramado sintético estariam associadas a maior incidência de lesões musculoesqueléticas, particularmente nos membros inferiores, em comparação com as disputadas em gramado natural.

## Materiais e Métodos

Neste estudo observacional e retrospectivo, foram analisados todos os 380 jogos da Série A do Campeonato Brasileiro de 2024, disputados entre abril e dezembro. As informações sobre o tipo de gramado utilizado em cada estádio foram obtidas nos *sites* oficiais das empresas responsáveis pela instalação e pela manutenção do gramado.

O site oficial da Confederação Brasileira de Futebol (CBF; https://www.cbf.com.br ) serviu como a principal fonte de dados na *web* , pois fornece relatórios de partidas, notificações de lesões, horários e informações relacionadas. Esses dados foram complementados por registros publicamente disponíveis nos principais meios de comunicação esportivos (Globo Esporte: https://ge.globo.com ; ESPN: www.espn.com.br ; e TNT Sports www.tntsports.com.br ) para obter detalhes adicionais sobre as características da lesão. A extração dos dados foi realizada com base em mais de uma fonte, sempre que possível, e os casos com informações inconsistentes ou conflitantes foram excluídos.

Como o estudo se baseou exclusivamente em dados secundários disponíveis publicamente, os relatórios de lesões dependiam de informações divulgadas por clubes e por fontes de mídia. Consequentemente, relatórios incompletos, subnotificação (particularmente de lesões menores) e erros de classificação diagnóstica não puderam ser descartados, e foram considerados limitações inerentes ao desenho do estudo. Não obtivemos acesso a registros médicos oficiais, nem a sistemas padronizados de vigilância de lesões.

A coleta de dados abrangeu todas as 380 partidas até a 38ª rodada, e incluiu as seguintes variáveis: ocorrência de lesão, mecanismo traumático ou não traumático, retirada da partida, exposição ao gramado sintético na partida anterior, número de partidas desde a última exposição ao gramado sintético, histórico de lesão relatada na mesma região anatômica, duração estimada da ausência e tipo de tratamento (conservador ou cirúrgico). Lesões musculoesqueléticas foram definidas operacionalmente como lesões que afetam músculos, tendões, ligamentos, articulações ou ossos, relatadas durante ou imediatamente após uma partida, e que resultaram em atenção médica ou em ausência da partida. Essa definição operacional foi adaptada de estruturas estabelecidas de vigilância de lesões propostas pela FIFA, pela União das Associações Europeias de Futebol (Union of European Football Associations, UEFA) e pelo Comitê Olímpico Internacional (COI), e reconhecemos que a falta de avaliação clínica direta e de relatórios padronizados impediu a adesão total aos critérios de consenso internacional. 21 22

A distribuição das partidas de acordo com o tipo de gramado foi a seguinte: gramado natural – 172 partidas em Celebration Bermuda (Sod Solutions, Inc), 29 em TifGrand Bermuda (Atlanta Sod Company), 43 em Tifway 419 Bermuda, 22 em esmeralda natural, e 59 em azevém perene; gramado artificial – 19 partidas em Coolplay (SYNLawn Australia/APT), 19 em Geofill (Italgreen S.p.A.) e 16 em elastômero termoplástico ( *thermoplastic elastometer* , TPE, em inglês).

As variáveis adicionais incluíram tempo de partida, temperatura ambiente no início, mecanismo de lesão, presença de trauma direto, adequação de calçados relatada, número de partidas disputadas sem exposição ao gramado sintético e abordagem de tratamento. A exposição durante a partida foi estimada presumindo-se 22 jogadores por partida e duração de 90 minutos, o que corresponde a 33 horas-jogador por partida. A incidência de lesões foi expressa como lesões por mil horas-jogador para explicar o desequilíbrio da exposição. Ao todo, 49 lesões foram excluídas das análises de tempo de recuperação devido à indisponibilidade de informações de retorno ao jogo.

Foram realizadas subanálises excluindo lesões com histórico relatado na mesma região anatômica e aquelas claramente associadas ao trauma direto, com o objetivo de descrever a incidência de lesões em condições não traumáticas e de primeiro evento.

### Análise estatística

A análise estatística foi realizada para estimar a incidência e a prevalência associadas a fatores que podem influenciar a ocorrência de lesões e ao tipo de gramado presente nas partidas analisadas. Nesse contexto, foram realizadas análises estatísticas para avaliar a associação entre o tipo de superfície de jogo e a incidência de lesões em partidas oficiais. A estatística descritiva foi utilizada para o cálculo das frequências absolutas e relativas. A incidência de lesões foi calculada como o número de lesões por mil horas-jogador para contabilizar o número desigual de partidas e o tempo de exposição entre os gramados natural e sintético. Além disso, o teste U de Mann-Whitney foi aplicado para avaliar a associação entre variáveis categóricas e escalares (não paramétricas, conforme confirmado pelo teste de Kolmogorov-Smirnov) de interesse, adotando-se um nível de significância estatística de *p* < 0,05.

Seguindo o mesmo nível de significância, utilizou-se o teste do Qui-quadrado (χ ^2^ ) para avaliar a associação entre variáveis categóricas de interesse. Além disso, as razões de chances (RCs) e as razões de prevalência (RPs), juntamente com seus respectivos ICs95%, foram calculadas para analisar as variáveis investigadas com a ocorrência de lesões e o tipo de tratamento adotado em resposta a potenciais fatores de risco. As razões de taxa de incidência (RTIs) e os ICs95% correspondentes foram estimados por meio de modelos de regressão de Poisson, com o *log* (hora-jogador) como variável de deslocamento.

A regressão logística não foi realizada porque os dados disponíveis foram agregados no nível da partida e não incluíram a exposição individual no nível do jogador, nem os dados de resultados binários necessários à modelagem de probabilidade válida. Além disso, nenhum ajuste multivariado foi realizado para potenciais fatores de confusão devido à falta de informações consistentes e confiáveis sobre essas variáveis em fontes de dados publicamente disponíveis.

Como este estudo incluiu todas as partidas oficiais da temporada sob investigação, nenhum tamanho de amostra *a priori* nem cálculo de poder estatístico foi realizado. As estimativas de efeito foram, portanto, interpretadas com base em intervalos de precisão e de confiança. Todas as análises foram realizadas no programa IBM SPSS Statistics for Windows (IBM Corp.), versão 23.0.

## Resultados

Este estudo analisou a Série A do Campeonato Brasileiro de Futebol, que incluiu 20 equipes, cada uma jogando 38 partidas (380 no total), de 13 de abril a 8 de dezembro de 2024. Os jogos foram realizados em 20 estádios: 3 com gramado sintético e 17 com gramado natural. Consequentemente, 3 equipes jogaram em casa em gramado sintético e 17, em gramado natural. Os gramados sintéticos incluíram TPE, Coolplay e Geofill, e os naturais incluíram Celebration Bermuda, TifGrand Bermuda, Tifway 419 Bermuda, esmeralda natural e azevém perene ( Tabela 1 ). A exposição durante a partida foi estimada presumindo-se 22 jogadores por partida e uma duração padrão de 90 minutos, o que corresponde a 33 horas-jogador por partida.

**Tabela 1:** Estádios com seus respectivos tipos de grama. Tabela criada usando o Microsoft Word (Microsoft Corp., Redmond, WA, EUA)

Estádio	Artificial	Tipo
**Estádio Artificial 1**	**SIM**	**Geofill**
Estádio Natural 1	NÃO	Bermuda Celebration
Estádio Natural 2	NÃO	Bermuda Celebration
Estádio Natural 3	NÃO	Bermuda Celebration
**Estádio Artificial 2**	**SIM**	**Cool Play**
Estádio Natural 4	NÃO	Bermuda Celebration
Estádio Natural 5	NO	Perennial Ryegrass
Estádio Natural 6	NÃO	Esmeralda Natural
Estádio Natural 7	NÃO	Bermuda Celebration
Estádio Natural 8	NÃO	Bermuda Tifgrand
Estádio Natural 9	NÃO	Bermuda Celebration
Estádio Natural 10	NÃO	Bermuda Celebration
Estádio Natural 11	NÃO	Bermuda Celebration
Estádio Natural 12	NÃO	Bermuda Tifgrand
Estádio Natural 13	NÃO	Perennial Ryegrass
Estádio Natural 14	NÃO	Perennial Ryegrass
**Estádio Natural 3**	**SIM**	**Thermoplastic (TPE)**
Estádio Natural 15	NÃO	Bermuda Celebration
Estádio Natural 16	NÃO	Bermuda Tifway 419
Estádio Natural 17	NÃO	Bermuda Tifway 419

### Análise Estatística

A incidência de lesões por tipo de gramado foi calculada como a razão entre as lesões registradas e o total de partidas disputadas em cada gramado. Frequências relativas, como as partidas no gramado Coolplay, foram usadas para avaliar a probabilidade de lesão por condição. Uma associação positiva, porém sutil, surgiu entre o tipo de gramado e o número de lesões (Mann-Whitney U = 7336; Z = -2,036; *p* = 0,042), o que indica um maior número de lesões por partida em gramado sintético, sem evidência estatisticamente significativa de causalidade. O histórico de lesões não se associou ao número de lesões por partida (Mann-Whitney U = 433; Z = -1,126; *p* = 0,260). A RP indicou que jogadores sem lesões prévias apresentaram efeito protetor (RP = 0,746; IC95% = 0,652–0,855).

Ao avaliar a ocorrência de lesão *versus* o tipo de gramado, não foi encontrada associação estatisticamente significativa (χ ^2^ (2) = 4,153; *p* = 0,054), embora a RC sugerisse um risco marginal (RC = 1,981; IC95% = 1,017–3,858; *p* = 0,042). Em relação ao tipo de gramado (natural *versus* sintético) e ao tratamento (conservador *versus* cirúrgico), não houve associação significativa (χ ^2^ (2) = 1,454; *p* = 0,257). A RC (3,214; IC95% = 0,387–26,727) sugeriu que o tratamento conservador foi mais frequente em gramado sintético, mas o amplo intervalo de confiança indicou inconclusividade.

A análise das lesões relacionadas ao trauma e do tipo de tratamento revelou uma tendência não significativa (χ ^2^ (2) = 3,164; *p* = 0,075). Embora a RC (0,325; IC95% = 0,09–1,168) tenha sugerido que o trauma poderia aumentar a probabilidade de tratamento cirúrgico, o intervalo amplo não confirmou significância estatística.

### Incidência de Lesões em Diferentes Tipos de Gramado: Natural e Sintético

**Fig. 1:**
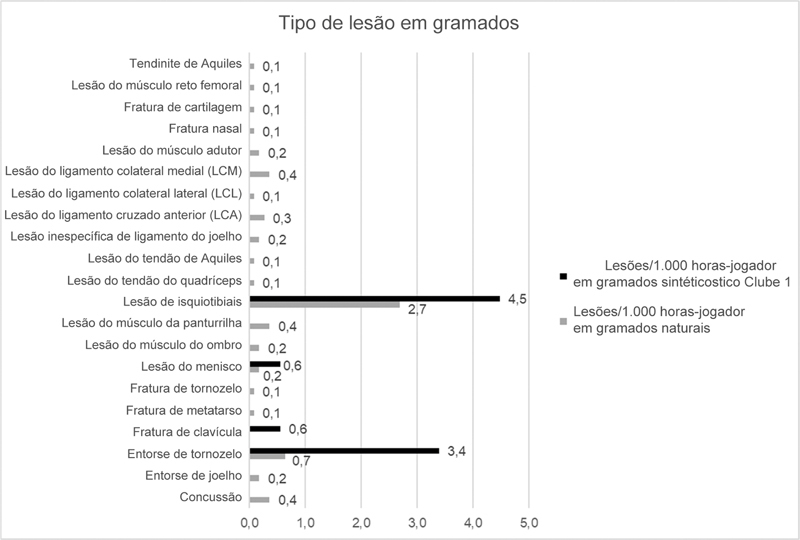
Tipo de lesão por mil horas-jogador em gramados naturais e sintéticos.

Um total de 88 lesões foi registrado, o que corresponde a uma média de 0,23 lesões por partida (IC95%: 0,17–0,28), sendo 71 em gramado natural em 326 partidas, e 17 em gramado sintético em 54 partidas. Quando ajustada pela exposição, a incidência de lesões isquiotibiais foi de 4,5 por mil horas-jogador em gramado sintético, e de 2,7 por mil horas-jogador em gramado natural ( Fig. 1 ). Quando normalizada pela exposição, a incidência foi de 6,6 por mil horas-jogador em gramado natural, e de 9,5 por mil horas-jogador em gramado sintético. A RTI entre os gramados sintético e natural foi de 1,44 (IC95%: 0,85–2,45; *p* = 0,18), embora o IC95% tenha incluído a unidade. A incidência de entorse de tornozelo foi de 3,4 lesões por mil horas-jogador em gramado sintético em comparação com 0,7 lesões por mil horas-jogador em gramado natural ( Fig. 1 ). Com base nas informações publicamente disponíveis sobre lesões relatadas anteriormente na mesma região anatômica, a diferença na incidência de entorse de tornozelo foi atenuada.

### Vantagem da Equipe da Casa: Gramados Sintético e Natural

**Fig. 2:**
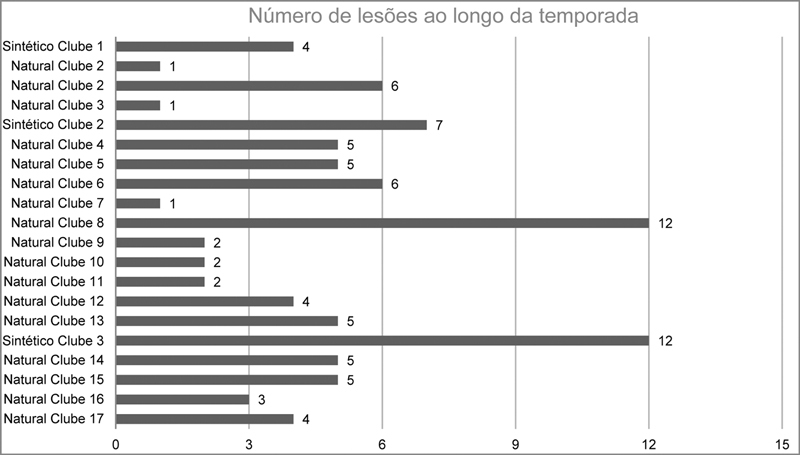
Número de lesões ao longo da temporada.

Duas equipes – uma cujo estádio era de gramado sintético, e outra, de gramado natural –registraram contagens de lesões idênticas (12 cada) ao longo da temporada, apesar dos diferentes perfis de exposição ( Fig. 2 ).

### Incidência de Lesões entre Tipos de Gramado Sintético

**Fig. 3:**
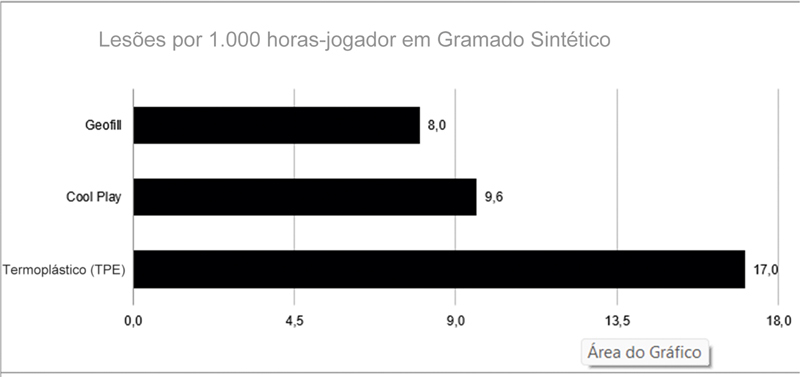
Lesões por mil horas-jogador por tipo de gramado sintético e natural.

**Fig. 4:**
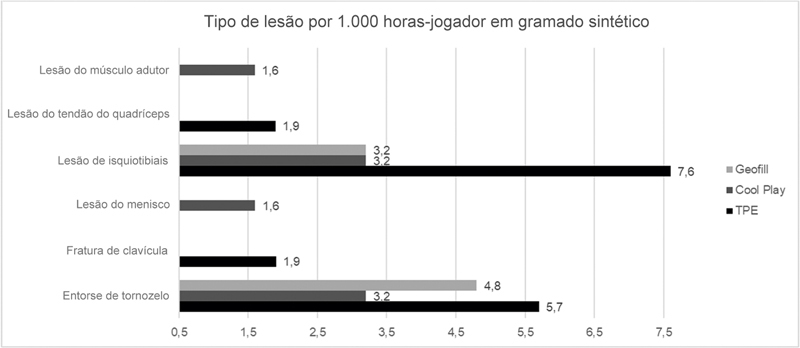
Tipo de lesão por mil horas-jogador em gramado sintético.

Ao todo, 16 partidas foram disputadas no gramado de TPE, o que resultou em uma incidência de 17 lesões por mil horas-jogador. O gramado Coolplay recebeu 19 partidas, com uma incidência de 9,6 lesões por mil horas-jogador, e o gramado Geofill também recebeu 19 partidas, com uma incidência de 8,0 lesões por mil horas-jogador ( Fig. 3 ). Após o ajuste para lesões anteriores e mecanismos de trauma direto, a incidência de lesões diminuiu para 11,4 por mil horas-jogador no TPE, 9,6 no Coolplay, e 6,4 no Geofill. O gramado de TPE apresentou maiores taxas de lesões musculares isquiotibiais (7,6) e de entorses de tornozelo (5,7). No gramado Coolplay, foi registrada uma taxa de lesões nos isquiotibiais e entorses de tornozelo de 3,2 por mil horas-jogador, ao passo que a taxa de lesões nos músculos adutores e lesões no menisco foi de 1,6 por mil horas-jogador. O gramado Geofill apresentou valores gerais mais baixos, com 4,8 entorses de tornozelo por mil horas-jogador e 3,2 lesões musculares isquiotibiais por mil horas-jogador, ao passo que outros tipos de lesões permaneceram com taxas abaixo de 2,0 por mil horas-jogador ( Fig. 4 ).

### Incidência de Lesões entre os Tipos de Gramado Natural

**Fig. 5:**
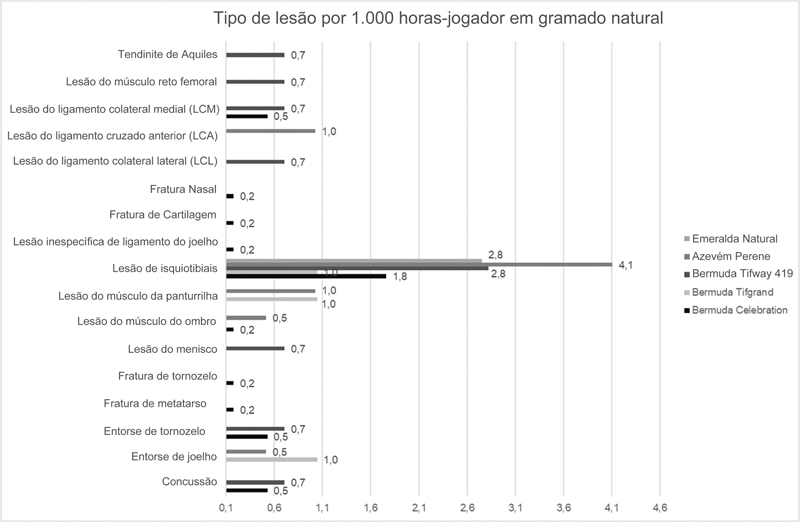
Tipo de lesão por mil horas-jogador em gramado natural.

Entre os tipos de gramado natural, o azevém perene apresentou 7,6 lesões por mil horas-jogador em 60 partidas. O Celebration Bermuda registrou 4,6 lesões por mil horas-jogador em 172 partidas, ao passo que o Tifway 419 apresentou 7,0 lesões por mil horas-jogador em 43 partidas. As taxas de incidência de lesões também foram observadas no TifGrand (3,1 lesões por mil horas-jogador em 29 partidas) e no esmeralda natural (2,8 lesões por mil horas-jogador em 22 partidas) ( Fig. 3 ). Após o ajuste por lesões anteriores e mecanismos de trauma, a incidência geral de lesões diminuiu. Quando as lesões em gramado natural são expressas por mil horas-jogador, as lesões posteriores da coxa apresentam os maiores valores, particularmente no azevém perene (4,1), seguido do esmeralda natural (2,8) e do Celebration Bermuda (2,0). As lesões na panturrilha variaram de 1,0 por mil horas-jogador em azevém perene a valores inferiores a 1,0 em outros gramados naturais. As lesões ligamentares do joelho foram registradas em valores absolutos baixos, com lesões do ligamento cruzado anterior atingindo 1,0 por mil horas-jogador no azevém perene, ao passo que as lesões dos ligamentos colaterais medial e lateral geralmente permaneceram em torno de 0,7 ou menos em todos os tipos de gramado natural. Outros tipos de lesões, incluindo lesões meniscais, entorses de tornozelo, fraturas, lesões no ombro e concussões, foram observados em baixas frequências, tipicamente entre 0,2 e 0,7 lesões por mil horas-jogador, com pequenas variações entre os diferentes gramados naturais ( Fig. 5 ).

### Horário da Partida e Condições Meteorológicas

**Fig. 6:**
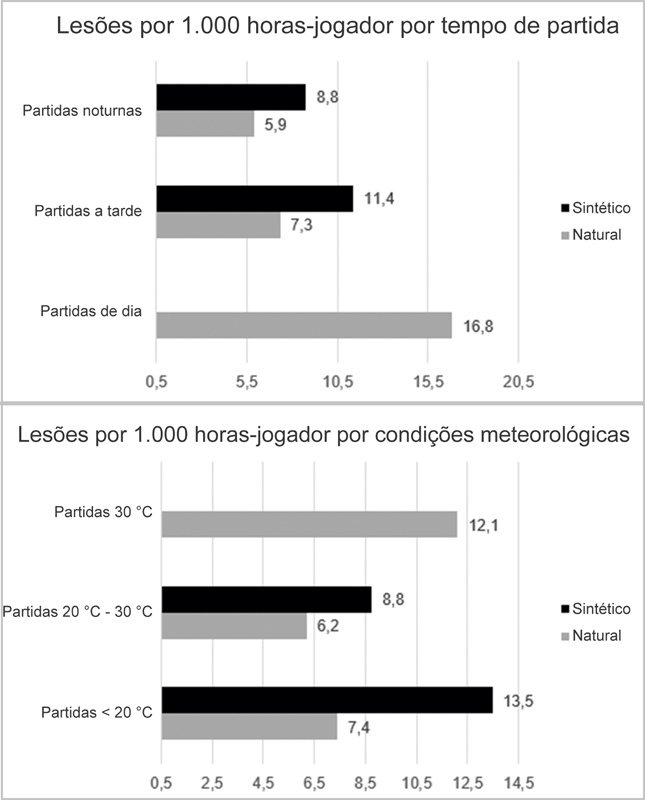
Lesões por mil horas-jogador por horário da partida e condições meteorológicas.

Quando a incidência de lesões foi descrita em função do horário da partida, observaram-se distribuições distintas entre os tipos de gramado. Nas partidas matinais, as lesões foram registradas somente nos gramados naturais, o que corresponde a uma incidência de 16,8 lesões por mil horas-jogador. Durante as partidas da tarde, a incidência de lesões em gramados sintéticos foi de 11,4 por mil horas-jogador, ao passo que, nos gramados naturais, a taxa foi de 7,3 por mil horas-jogador. Em partidas noturnas, a incidência de lesões foi de 8,8 por mil horas-jogador nos gramados sintéticos, e de 5,9 por mil horas-jogador nos gramados naturais. Em relação às condições climáticas, a incidência de lesões variou conforme as faixas de temperatura e os tipos de gramado. Em partidas realizadas abaixo de 20 °C, foram observados valores de incidência de lesões de 13,5 por mil horas-jogador nos gramados sintéticos, e de 7,4 por mil horas-jogador nos gramados naturais. Entre 20 °C e 30 °C, a incidência de lesões foi de 8,8 por mil horas-jogador nos gramados sintéticos, e de 6,2 por mil horas-jogador nos gramados naturais. Em partidas disputadas em temperaturas acima de 30 °C, somente foram registradas lesões nos gramados naturais, com uma incidência de 12,1 por mil horas-jogador, pois nenhuma partida em gramado sintético ocorreu nessas condições ( Fig. 6 ).

### Tipo de Tratamento e Tempo de Recuperação

Das 71 lesões sofridas em 326 partidas nos gramados naturais, 12 corresponderam a uma incidência de tratamento cirúrgico de 1,1 cirurgias por mil horas-jogador, ao passo 59 resultaram em uma incidência de tratamento conservador de 5,5 por mil horas-jogador. Nos gramados sintéticos, 17 lesões ocorreram ao longo de 54 partidas, com a incidência de tratamento cirúrgico de 0,6 cirurgias por mil horas-jogador. O tempo de recuperação geral relacionado à lesão não diferiu significativamente entre os gramados (diferença média = 0,43 semanas; *p* > 0,05), com diferença média de 0,43 semanas (aproximadamente 4 dias), sendo ligeiramente maior nas lesões nos gramados naturais.

No geral, a análise revelou padrões consistentes. As lesões dos membros inferiores predominaram, independentemente do tipo de gramado, com distensões isquiotibiais e entorses de tornozelo sendo as mais frequentemente observadas. O gramado sintético tendeu a apresentar maior incidência de lesões por partida, particularmente lesões musculares e relacionadas ao tornozelo, embora as diferenças entre os gramados tenham sido geralmente pequenas após a exposição à normalização. Entre os subtipos de gramado, a maior variabilidade foi observada em gramados sintéticos específicos e em azevém perene, entre os gramados naturais. Análises temporais e ambientais indicaram maior incidência de lesões durante as partidas da tarde e da noite e sob temperaturas mais frias em gramado sintético, ao passo que temperaturas mais altas foram associadas ao aumento da incidência de lesões em gramado natural. Coletivamente, os dados destacam tendências específicas do gramado influenciadas pela exposição, pela temperatura e pelas condições de correspondência, sem demonstrar disparidades grandes ou consistentes entre os tipos de gramado.

## Discussão

Este estudo fornece uma análise epidemiológica das taxas de lesões no futebol profissional masculino brasileiro e demonstra que, após a normalização da exposição, as diferenças entre gramados sintéticos e naturais foram geralmente pequenas, apesar da presença de alguns padrões específicos dos gramados. Na Série A do Campeonato Brasileiro de 2024, a incidência de lesões por mil horas-jogador foi numericamente maior nos gramados sintéticos do que nos naturais (9,5 *versus* 6,6, respectivamente), sem evidências estatisticamente significativas de relação causal. A associação observada pode refletir características do gramado; no entanto, o desenho retrospectivo do estudo não permite inferências mecanicistas ou causais.

Em contraste, Kuitunen et al. 8 relataram uma incidência de lesões 21% menor no gramado sintético entre jogadores profissionais. Esses achados, no entanto, podem não ser diretamente comparáveis aos presentes resultados devido a diferenças no contexto competitivo, nos sistemas de vigilância de lesões, na padronização de gramados e nas demandas biomecânicas específicas do esporte.

Entre as 3 equipes que jogaram partidas em casa em gramado sintético, somente 1 apresentou incidência de lesões acima da média da liga, com 17 lesões por mil horas-jogador, sendo 7,6 nos isquiotibiais e 5,7 entorses de tornozelo. Esta equipe jogou em gramado TPE, o único gramado deste tipo entre os estádios analisados, ao passo que os gramados Coolplay e Geofill apresentaram taxas de incidência semelhantes. Esses achados sugerem que a variabilidade nos gramados sintéticos pode ser influenciada por múltiplos fatores contextuais, incluindo características de gramado, qualidade de manutenção, carga de treinamento, histórico clínico dos jogadores, estilo de jogo e condições ambientais durante as partidas. 8 13 15

Consistente com os resultados, o gramado sintético, particularmente o de TPE, mostrou maior incidência de lesões específicas, notadamente lesões musculares da coxa e entorses de tornozelo. As lesões musculares da coxa foram predominantemente distensões dos isquiotibiais, com uma incidência de 4,5 lesões por mil horas-jogador em gramado sintético em comparação com 2,7 em gramado natural. As lesões no tornozelo, particularmente as entorses, apresentaram a maior discrepância entre os gramados, com incidência de 3,4 por mil horas-jogador em gramado sintético contra 0,7 em gramado natural, o que concorda com a literatura anterior. 4 6 23 No entanto, essa diferença foi atenuada após a exclusão de lesões associadas a trauma direto e de lesões prévias relatadas na mesma região anatômica.

Os mecanismos mais comuns associados às lesões no tornozelo incluem inversão, eversão e estresse rotacional, potencialmente influenciados pela interação entre o calçado e o tipo de gramado. Em contrapartida, lesões envolvendo outras regiões, como quadril e joelho, não demonstraram diferenças relevantes na incidência na comparação entre os gramados. Deve-se notar também que o número substancialmente menor de partidas disputadas em gramado sintético (54 *versus* 326) pode ter limitado a detecção de lesões menos frequentes e influenciado os padrões de distribuição de lesões. 2 6 23 24

Fatores adicionais, como o clima regional, o uso não esportivo de estádios, a frequência e a qualidade de manutenção e os curtos intervalos de recuperação entre as partidas, podem afetar significativamente as condições do campo e, consequentemente, o risco de lesões. Comparações mais robustas entre gramados exigiriam padrões de manutenção e condições de jogo equivalentes, que são difíceis de alcançar em ambientes reais de futebol profissional. 23

Em relação ao tempo de jogo, a maioria das lesões ocorreu durante as partidas da tarde e da noite, especialmente em gramado sintético. O pequeno número de partidas matinais, com apenas cinco lesões registradas em gramado natural e nenhuma em gramado sintético, limita as comparações diretas entre os períodos. As partidas da manhã apresentaram maior incidência de lesões no gramado natural, ao passo que as partidas da tarde e da noite apresentaram maior incidência em gramados sintéticos, o que sugere que o risco de lesão pode variar de acordo com o tipo de gramado e o horário da partida.

A temperatura ambiente também influenciou a incidência de lesões. Em partidas realizadas abaixo de 20 °C, a incidência de lesões foi maior no gramado sintético (13,5 *versus* 7,4 lesões por mil horas-jogador). Por outro lado, temperaturas mais altas associaram-se ao aumento da incidência de lesões no gramado natural. Esses achados diferem de estudos no futebol americano universitário, em que temperaturas mais baixas foram associadas a taxas reduzidas de lesões em gramado sintético, o que indica que a relação entre temperatura, tipo de gramado e risco de lesão depende do esporte e do contexto. 15

Com relação ao tratamento, as lesões no gramado sintético foram mais frequentemente tratadas de forma conservadora, ao passo que as no gramado natural foram mais frequentemente associadas à intervenção cirúrgica. No entanto, a modalidade de tratamento por si só não reflete necessariamente a gravidade da lesão, e esses achados devem ser interpretados com cautela, dada a natureza descritiva dos dados.

Finalmente, as características individuais do atleta, juntamente com a qualidade e a manutenção do gramado, continuam sendo os principais determinantes do risco de lesões e da recuperação. Consequentemente, os achados deste estudo devem ser interpretados como descritivos e exploratórios, pois fornecem uma visão geral da ocorrência e da distribuição de lesões entre os tipos de gramado, sem apoiar conclusões definitivas quanto à causalidade ou à prevalência de lesões relacionadas ao tipo de gramado. 15

### Limitações

Uma limitação importante deste estudo está relacionada ao método de coleta de dados de lesões, que se baseou exclusivamente em fontes secundárias publicamente disponíveis, incluindo comunicações oficiais dos clubes e relatórios da mídia. Embora essas fontes permitam uma visão geral ampla da ocorrência de lesões ao longo da temporada, a ausência de um sistema padronizado de vigilância de lesões e de avaliação clínica direta pode comprometer a precisão das informações sobre o diagnóstico, a gravidade, o mecanismo e o manejo terapêutico da lesão. Consequentemente, a subnotificação (particularmente de lesões menores), a classificação incorreta e a heterogeneidade nas definições de lesões entre as fontes não podem ser descartadas e representam limitações inerentes ao desenho do estudo.

Embora os sistemas internacionais de vigilância de lesões propostos pela FIFA, UEFA e pelo COI forneçam orientação metodológica padronizada, sua aplicação completa não foi viável devido à dependência exclusiva de fontes de dados secundárias disponíveis publicamente.

Além disso, as informações de retorno ao jogo não estavam disponíveis para aproximadamente 38% das lesões relatadas, o que limitou a robustez das análises sobre o tempo de recuperação e a gravidade da lesão. Essa limitação reflete ainda mais a dependência da integridade e da precisão dos dados relatados publicamente.

Outra limitação relevante diz respeito ao desequilíbrio de exposição. A maioria das partidas foi disputada em gramado natural, pois apenas 3 dos 20 estádios da liga usavam gramado sintético. Como resultado, 54 partidas foram disputadas em gramados sintéticos, em comparação com mais de 300 em gramado natural, o que pode ter reduzido o poder estatístico para detectar diferenças, particularmente em tipos de lesões menos frequentes.

Além disso, a população do estudo foi restrita a jogadores profissionais de futebol masculino que competem na Série A brasileira. Portanto, os resultados podem não ser generalizáveis para jogadores do sexo feminino, jovens, de nível amador ou atletas de outros esportes praticados em gramado sintético.

A falta de controle sobre vários fatores de confusão potenciais representa uma limitação adicional. Variáveis como carga de treinamento, estilo de jogo, calçados, histórico detalhado de lesões anteriores, desgaste do campo ao longo da temporada, práticas de manutenção e diferenças estruturais ou climáticas entre estádios não puderam ser sistematicamente controladas, e podem ter influenciado os padrões de lesões observados.

Finalmente, vários parâmetros explorados neste estudo, como vantagem em casa, tempo de jogo, temperatura ambiente e análises estratificadas por sistemas específicos de gramado sintético, permanecem pouco investigados na literatura. Essa escassez limita as comparações diretas e a interpretação contextual mais aprofundada dos achados. Estudos prospectivos, utilizando sistemas padronizados de vigilância de lesões e de controle abrangente de variáveis de confusão, são necessários para confirmar e ampliar essas observações.

## Conclusão

No contexto da Série A do Campeonato Brasileiro, uma incidência maior de lesões musculoesqueléticas foi observada em gramado sintético, em comparação com gramado natural, com distensões isquiotibiais mais comuns em gramado natural e entorses de tornozelo mais frequentes em gramado sintético. Em geral, as diferenças na ocorrência de lesões entre os tipos de gramado foram mínimas.

A frequência de lesões também foi associada às condições climáticas, com maior ocorrência em gramado sintético em temperaturas mais baixas e em gramado natural em temperaturas mais altas. Lesões que requerem tratamento cirúrgico foram mais frequentemente relatadas em gramado natural, embora as diferenças de tempo de recuperação na comparação entre os gramados fossem pequenas.

Esses achados representam associações observacionais e devem ser interpretados com cautela devido às limitações metodológicas, incluindo a dependência de dados secundários e a exposição desequilibrada, e não permitem conclusões definitivas sobre a prevalência de lesões por tipo de gramado. Estudos prospectivos com vigilância padronizada de lesões são necessários.

## References

[1] Calloway S P Hardin D M Crawford M D Injury Surveillance in Major League Soccer: A 4-Year Comparison of Injury on Natural Grass Versus Artificial Turf Field Am J Sports Med 2019 47 10 2279 2286 10.1177/0363546519860522 31306590

[2] Gosnell G G Gerber B A Guyton G P Gould H P Playing Surface and Injury Risk: Artificial Turf vs. Natural GrassIn: Wojda TR, Stawicki SP, editors. Injuries and Sports Medicine Londres: IntechOpen;2022 10.5772/intechopen.106615

[3] Mack C D Hershman E B Anderson R B Higher Rates of Lower Extremity Injury on Synthetic Turf Compared With Natural Turf Among National Football League Athletes: Epidemiologic Confirmation of a Biomechanical Hypothesis Am J Sports Med 2019 47 01 189 196 10.1177/0363546518808499 30452873

[4] Ammar A Bailey S J Hammouda O Effects of Playing Surface on Physical, Physiological, and Perceptual Responses to a Repeated-Sprint Ability Test: Natural Grass Versus Artificial Turf Int J Sports Physiol Perform 2019 14 09 1219 1226 10.1123/ijspp.2018-0766 30860407

[5] Bianco A Spedicato M Petrucci M A Prospective Analysis of the Injury Incidence of Young Male Professional Football Players on Artificial Turf Asian J Sports Med 2016 7 01 e28425 10.5812/asjsm.28425 27217929 PMC4870829

[6] Gould H P Lostetter S J Samuelson E R Guyton G P Lower Extremity Injury Rates on Artificial Turf Versus Natural Grass Playing Surfaces: A Systematic Review Am J Sports Med 2023 51 06 1615 1621 10.1177/03635465211069562 35593739

[7] Kent R Yoder J O'Cain C M Force-limiting and the mechanical response of natural turfgrass used in the National Football League: A step toward the elimination of differential lower limb injury risk on synthetic turf J Biomech 2021 127 110670 10.1016/j.jbiomech.2021.110670 34391130

[8] Kuitunen I Immonen V Pakarinen O Mattila V M Ponkilainen V T Incidence of football injuries sustained on artificial turf compared to grass and other playing surfaces: a systematic review and meta-analysis EClinicalMedicine 2023 59 101956 10.1016/j.eclinm.2023.101956 37125402 PMC10139885

[9] Lanzetti R M Ciompi A Lupariello D Guzzini M De Carli A Ferretti A Safety of third-generation artificial turf in male elite professional soccer players in Italian major league Scand J Med Sci Sports 2017 27 04 435 439 10.1111/sms.12654 26888457

[10] Meyers M C Incidence, Mechanisms, and Severity of Match-Related Collegiate Men's Soccer Injuries on FieldTurf and Natural Grass Surfaces: A 6-Year Prospective Study Am J Sports Med 2017 45 03 708 718 10.1177/0363546516671715 27872124

[11] Lessley D J Kent R W Funk J R Video Analysis of Reported Concussion Events in the National Football League During the 2015-2016 and 2016-2017 Seasons Am J Sports Med 2018 46 14 3502 3510 10.1177/0363546518804498 30398897

[12] Loughran G J Vulpis C T Murphy J P Incidence of Knee Injuries on Artificial Turf Versus Natural Grass in National Collegiate Athletic Association American Football: 2004-2005 Through 2013-2014 Seasons Am J Sports Med 2019 47 06 1294 1301 10.1177/0363546519833925 30995074

[13] Meyers M C Incidence, Mechanisms, and Severity of Game-Related High School Football Injuries Across Artificial Turf Systems of Various Infill Weights Orthop J Sports Med 2019 7 03 2.325967119832878E15 10.1177/2325967119832878 PMC6434442 30937317

[14] Nunes H EG Onaka G M Gaspar J J Junior Barbosa F SS Martinez P F Oliveira S A Júnior Prevalência e fatores associados às lesões esportivas em jovens jogadores de futebol Arch Health Sci 2021 28 01 34 37 10.17696/2318-3691.28.1.2021.1927

[15] Paliobeis A Sivasundaram L Knapik D M Injury incidence is higher on artificial turf analyzed with natural grass in high school athletes: a retrospective cohort study Curr Orthop Pract 2021 32 04 355 360 10.1097/bco.0000000000001012

[16] Ataabadi Y A Sadeghi H Ataabadi M H The Effects of Artificial Turf on the Performance of Soccer Players and Evaluating the Risk Factors Analyzed to Natural Grass J Neurol Res Ther 2017 2 05 1 16 10.14302/issn.2470-5020.jnrt-17-1487

[17] Sivasundaram L Mengers S Paliobeis A Injury risk among athletes on artificial turf: a review of current literature Curr Orthop Pract 2021 32 05 512 517 10.1097/BCO.0000000000001021

[18] Twomey D M Petrass L A Fleming P Lenehan K Abrasion injuries on artificial turf: A systematic review J Sci Med Sport 2019 22 05 550 556 10.1016/j.jsams.2018.11.005 30503328

[19] Wannop J W Foreman T Madden R Stefanyshyn D Influence of the composition of artificial turf on rotational traction and athlete biomechanics J Sports Sci 2019 37 16 1849 1856 10.1080/02640414.2019.1598923 30922172

[20] Xiao M Lemos J L Hwang C E Sherman S L Safran M R Abrams G D Increased Risk of ACL Injury for Female but Not Male Soccer Players on Artificial Turf Versus Natural Grass: A Systematic Review and Meta-Analysis Orthop J Sports Med 2022 10 08):23259671221114353 10.1177/23259671221114353 PMC9382072 35990873

[21] Fuller C W Ekstrand J Junge A Consensus statement on injury definitions and data collection procedures in studies of football (soccer) injuries Br J Sports Med 2006 40 03 193 201 10.1136/bjsm.2005.025352 16505073 PMC2491990

[22] Bahr R Clarsen B Derman W International Olympic Committee consensus statement: methods for recording and reporting of epidemiological data on injury and illness in sport 2020 (including STROBE Extension for Sport Injury and Illness Surveillance (STROBE-SIIS)) Br J Sports Med 2020 54 07 372 389 10.1136/bjsports-2019-101969 32071062 PMC7146946

[23] De Lunetta A Silva A V Souza F Grama natural vs. grama sintética: qual é a melhor opção para os estádios? Rev Acad Lusofonia 2024 1 03 1 10 10.5281/zenodo.13826563

[24] Zucolotto T E Gerônimo R MP Silva P IJ da Costa L CS Lesões ligamentares do tornozelo em atletas: prevenção e tratamento Braz J Heath Rev 2023 6 06 31313 31324 10.34119/bjhrv6n6-365

